# The Mediating Role of Cognitive Flexibility in the Influence of Counter-Stereotypes on Creativity

**DOI:** 10.3389/fpsyg.2019.00105

**Published:** 2019-02-05

**Authors:** Bin Zuo, Fangfang Wen, Miao Wang, Yang Wang

**Affiliations:** ^1^School of Psychology, Central China Normal University, Wuhan, China; ^2^Center for Studies of Social Psychology, Central China Normal University, Wuhan, China; ^3^Key Laboratory of Adolescent Cyberpsychology and Behavior, Ministry of Education, Wuhan, China

**Keywords:** counter-stereotypes, creativity, cognition flexibility, meditating effect, emotion

## Abstract

The aim of this study is to explore the relationship between counter-stereotypes and creativity, and further explore the mechanism underlying the impact of priming counter-stereotypic information on individual creativity. More importantly, here we have proposed cognitive and emotional dual processing pathways, which may mediate the influences of counter-stereotypes on creativity. Two experiments examined how counter-stereotypes impacted creativity through the dual processing pathways. A total of 152 university students were recruited to test their creativity performance. In Experiment 1, we replicated results of past studies. Participants were randomly allocated to different priming conditions (stereotype or counter-stereotype), in which descriptions of male governors and female nurses served as priming of stereotypes, whereas descriptions of male nurses and female governors served as priming of counter-stereotypes. Measurements of creativity were based on the poster paradigm. The poster paradigm required participants to design a poster for a college fellowship party. In Experiment 2, we recruited 104 participants to examine the mediating roles of emotions and cognitive flexibility. The procedure of Experiment 2 was similar to that of Experiment 1, except for the measurement of creativity, which was Chinese idiom riddle test. Participants who selected more creative answers were more creative, based on the criteria of our experimental design. Also, we included measurements of emotions (i.e., surprise and delight) and cognitive flexibility (using the Cognitive Flexibility Scale) after priming of stereotypes and counter-stereotypes in Experiment 2. We also verified the credibility of our counter-stereotype measurements. The results of Experiment 1—which replicated previous studies—demonstrated that priming of counter-stereotypes promoted creative performance compared with priming of stereotypes in the poster paradigm. However, our proposed dual processing pathways were not fully verified by Experiment 2. The results of this experiment showed that neither surprising nor delighted emotion mediated the influence of counter-stereotypes on creativity, whereas cognitive flexibility did. In conclusion, our current study reveals a mechanism of creative performance in terms of cognitive flexibility, and further inspires us to focus on the positive influence of counter-stereotypes on creativity.

## Introduction

Creative ability has played an important role in the development of human society (Gong et al., 2016). Creativity is considered as a primary motivational factor and a core competency for the development of enterprises, organizations, and nations. Similarly, creativity is an indispensable quality for individual development and is in greater demand in modern-day China. Thus, more research is required to develop methods for increasing creativity. On the other hand, China is a collectivistic country (Hofstede, 2001), and the environment here is a disadvantage of personal creativity development comparing with individualistic countries (Goncalo and Staw, 2006; Zha et al., 2006). Former results about creativity promotion method might present differently for collectivism culture. Therefore, it is also necessary to reexamine the effectiveness of creativity training in China. Previously, psychologists have widely explored methods of innovation and creativity training. As early as 1950, JP Guilford had already advocated research in the area of creativity, which then increased the number of researchers focused on its structural components. Today, research on creativity is continuously developing, improving its measurements (Hocevar, 1981; Kim, 2006), training people’s creativity (Scott et al., 2004), better understanding its social and cultural influences (Amabile, 1983; Shalley and Gilson, 2004), and exploring its unique cognitive neural mechanisms (Dietrich and Kanso, 2010; Beaty et al., 2016).

Recently, social psychologists have conducted extensive research on factors influencing individual creativity. This research has demonstrated that emotion and cognitive factors both play significant roles in individual creative performance (Ward, 2007; Akinola and Mendes, 2008; De Dreu et al., 2008). Furthermore, from the perspective of social cognition, several scholars have also found a close connection between stereotypes and creativity (Gocłowska et al., 2013). The present study focuses on the relationship between counter-stereotypes and creativity—based on previous research—and further examines the mechanism of counter-stereotypic priming on individual creativity performance in terms of cognitive and emotional factors.

Since the publication of Galton’s *Hereditary Genius* in 1869, researchers have constructed a variety of theories on creativity (Huang et al., 2005). Cognitive processing is regarded as an important factor that affects individual creative performance (Hayes, 1989; Batey and Furnham, 2006; Sternberg, 2006), as well as the degree of one’s independence, innovation, and flexibility. Many empirical studies have focused on cognitive factors influencing an individual’s creativity (Barron and Harrington, 1981; Simonton, 2014; Kandler et al., 2016). For example, Nusbaum and Silvia (2011) found modest correlations between cognitive executive processes and creativity. Next, using multivariate structural equation modeling, recent study further confirmed that both associative and executive processes have a significant impact on the production of novel ideas (Beaty et al., 2014). Furthermore, a subsequent functional Magnetic Resonance Imaging fMRI study revealed the inner-cognitive neural-activation mode of creative thought. A distributed network involving dorsolateral prefrontal cortex—a locus for cognitive executive networks—was found to support this process of creative thought (Beaty et al., 2015). In conclusion, studies have already found a close relationship between cognitive abilities and creativity.

Stereotypes are general and fixed cognitive views of a social group (Zhang et al., 2016; Sun et al., 2016; Song and Zuo, 2016). On the contrary, counter-stereotypes refer to an individual’s cognitive views of a social group—in terms of perceived behaviors or traits—which are inconsistent with or contrary to the mindset of the social group in question (Liu and Zuo, 2006; Leicht et al., 2017). Recently, many studies have focused on whether presentation of counter-stereotypic information would influence people’s cognitive abilities (Damer et al., 2017; Colombo et al., 2018). These studies have suggested that presentation of counter-stereotypic information not only reduces stereotypes and prejudice related to certain groups (Dasgupta and Greenwald, 2001; Columb and Plant, 2011; Lai et al., 2014; Finnegan et al., 2015), but also promotes an individual’s cognitive flexibilities and may concomitantly affect creativity (Asgari et al., 2010; Asgari et al., 2012). Therefore, theoretically, it seems that counter-stereotypic information could affect creativity performance through cognitive pathways.

Several researchers have attempted to examine the influence of counter-stereotypes on creativity directly (Parish and Hudson, 1970; Dumas and Dunbar, 2016). After activating participants’ stereotypes through an imagination of an “eccentric poet” or “rigid librarian,” researchers conducted divergent thinking tasks to measure participants’ creative performance. The results showed that stereotype activation could truly enhance participant’s divergent thinking abilities. Furthermore, in view of possible interferences of emotion, some other researches controlled these emotional variables and examined whether priming of counter-stereotypes also have similar effects (Gocłowska et al., 2013, 2014). These studies found that counter-stereotypes could increase cognitive flexibility while improving creativity performance. Cognitive flexibility refers to a kind of strategy or capability for flexible switching from one stimulation, manipulation, or psychological mode to another when necessary (Vartanian, 2009). The interpretation by the researchers was that, after activating counter-stereotypic information, the specific content of stereotypic knowledge was no longer effective. Participants would think more about other possibilities (i.e., exhibit more flexible thinking) and thereby increased their creativity.

Although existing studies have demonstrated the promotion of counter-stereotypes on individuals’ cognitive flexibility and creative performance, they have not considered possible mediating roles of cognitive flexibility among them. It is adaptive, as it helps people change their behavioral patterns and strategies effectively when facing new circumstances or environments, in order to solve problems (Heilman et al., 2003; Sligte et al., 2011). Presentation of counter-stereotype information is beneficial to improve an individual’s cognitive flexibility (Gocłowska et al., 2013), which is closely related to divergent thinking and creativity (Evans and Stanovich, 2013; Barr et al., 2015). Thus, cognitive flexibility may be an important mediating variable between counter-stereotypes and creativity.

Furthermore, counter-stereotypes may also affect creativity through emotions. Researchers have believed that counter-stereotypic information generates surprise, which interrupts an individual’s existing thinking process and diverts their attention to unexpected stimuli (Prati et al., 2015). Meanwhile, surprise may motivate people to analyze differences between cognitive schemas, which evoke curiosity regarding the nature of these differences. Some researchers have pointed out that high-activation positive emotions—such as those found to be exciting, energetic, and interesting—can improve creativity and lead people to perform better on insight tests and divergent thinking tasks (Ashby and Isen, 1999; Hirt et al., 2008; Conner and Silvia, 2015). Therefore, it seems plausible that emotional responses triggered by counter-stereotypes could enhance individual creativity as well.

As for the manipulation of stereotype priming, we selected stereotype and counter-stereotype priming based on previous studies (Gocłowska et al., 2013, 2014), which used gendered or racial exemplars for stereotype or counter-stereotype priming. However, racial cues are not predominant cues for Chinese, even for children (Zhang et al., 2018), so we only took gendered stereotype/counter-stereotype exemplars into account for different priming conditions.

Studies of gender stereotypes are often intertwined with occupational stereotypes (Eagly and Steffen, 1984; White and White, 2006; Bolukbasi et al., 2016). Assertiveness and performance indicate greater agency in men, while warmth and care for others are signs of greater communality in women; these gender biases lead to different occupational selections (Ellemers, 2018). Thus, the priming of our study focused on occupational gender stereotypes as an exemplar, where we selected governing as a high-agency occupation and nursing as a high-communal occupation. We chose male governors and female nurses as stereotype-priming exemplars, while female governors and male nurses were selected as counter-stereotype-priming exemplars. On the other hand, previous research has shown that people, regardless of their own gender, are less tolerant of men behaving in counter-stereotypic ways compared with such behavior in women (Signorella and Liben, 1984; Hughes and Seta, 2003; Sullivan et al., 2018). These findings imply that the promotion of creativity in terms of counter-stereotypes priming may be differed across target’s gender. Therefore, the influence of a target’s gender is also included in our analysis.

In summary, even though previous studies have illustrated a direct relationship between counter-stereotypes and creativity (Gocłowska et al., 2013, 2014), its mechanism has not been fully examined. Based on previous research, we argue that emotion and cognitive flexibility may both play roles in this process. Thus, the aim of this study is to replicate prior research using the poster paradigm (Gocłowska et al., 2013), as well as via a different paradigm based on Chinese culture. More importantly, this study proposes a two-pathway model to explain the mechanism of counter-stereotypes influencing the promotion of an individual’s creativity. This two-pathway model posits that counter-stereotypes affect creativity through emotion and cognitive flexibility; in other words, we hypothesize that emotion and cognitive flexibility play mediating roles in this process.

## Experiment 1: Influence of Counter-Stereotypes on Creativity

### Methods

#### Participants

There were 48 voluntary participants (24 males) involved in this experiment, *M_age_* = 19.17, *SD* = 1.99. Each participant was randomly arranged to one of the experiment conditions. The specific grouping and age distribution are shown in Table 1.

**Table 1:** Participants’ allocation and their age distribution in Experiment 1.

	Stereotype priming group			Counter-stereotype priming group		
Targets’ gender	*N*	*M_age_*	*SD*	*N*	*M_age_*	*SD*
Male	12	18.83	2.17	12	18.83	1.12
Female	12	19.67	1.78	12	19.33	2.71


This study was carried out in accordance with the recommendations of American Psychological Association (APA) ethical guidelines. The protocol was approved by the Ethics Committee of the Center for Studies of Social Psychology at Central China Normal University. Before conducting the formal experimental procedure, all participants were given an informed consent form in accordance with the Declaration of Helsinki. The informed consent form included a brief description about our study and some possible uncomfortable situations, as well as the confidentiality of their data in terms of remaining anonymous in any publication related to this study. It also informed them about their rights to withdrawal from the experiment at any time, and also included contact information of the researchers so that participants could inquire about any further details of the study. Participants indicated their willingness by checking the “I agree” option and signed their names. The informed consent procedure was identical for all following experiments.

### Materials

We recruited 37 participants (21 males, *M_age_* = 20.73, *SD* = 2.16) to examine the reliability of priming exemplars used in previous studies (i.e., male governor, male nurse; female governor, and female nurse). Participants were required to assess the typicality of four exemplars through the Likert 7-point scale (1 = very typical, 7 = very untypical), where higher scores indicate more counter-stereotypic exemplars. A repeated measure Analysis of Variance (ANOVA) showed that scores of counter-stereotype exemplars (*M* = 4.76, *SD* = 4.35) were significantly higher than scores of stereotype exemplars (*M* = 2.41, *SD* = 2.54), *p_s_* < 0.001. Therefore, these exemplars can be used for stereotype/counter-stereotype priming.

### Procedures

This experiment adopted a 2 × 2 randomized block design. The independent variables were targets’ gender (male vs. female) and priming type (stereotype priming vs. counter-stereotype priming). The dependent variable was their creativity on the poster design.

The procedures were conducted in our laboratory, and each participant completed the experiment alone. Participants were randomly assigned to one of our priming groups (male stereotype: male governors; male counter-stereotype: male nurses; female stereotype: female nurses; and female counter-stereotype: female governors). First, for stereotype/counter-stereotype priming, participants needed to complete a description task (Leicht et al., 2014). They were instructed to describe their corresponding group target with six different adjectives. Then, to test the effectiveness of the priming manipulation, participants also evaluated the typicality of the target using the 7-point Likert scale (1 = very typical, 7 = very atypical), which is identical with the procedure used in previous studies. Participants then had to answer what their perceived typicality was for each target in the target’s gender group. For example, if a participant were arranged into the male stereotype group, they needed to answer the question, “What is your perceived typicality of male governors based on male stereotypes?”.

To replicate the findings from previous studies, we also used the poster paradigm—which these studies used—to first measure participants’ creativity (Gocłowska et al., 2013). After the priming of stereotypes or counter-stereotypes, participants were asked to design a poster for their fellowship party, which needed to be as novel and unique as possible. Participants could draw their own poster in any form which they preferred within 5 min.

### Results

We used Statistical Package for the Social Sciences (SPSS) 21.0 to analyze our data. Before starting, three psychology postgraduate students were invited to evaluate the creativity of participants’ poster designs on a 7-point Likert scale. The postgraduate students were blinded to the design of our experiment and they made their evaluations individually on separate rating sheets. Once obtained, we calculated their internal consistency reliability, with Cronbach *α* = 0. 772, which indicated the reliability of their evaluation. Thus, we averaged their ratings of participants’ creativity of poster designs.

Firstly, we conducted an independent sample *t*-test to analyze the effectiveness of priming manipulation. The typicality of targets in stereotype priming groups was significantly lower than that of the counter-stereotype priming groups, which indicated an effective manipulation of priming.

**FIGURE 1:**
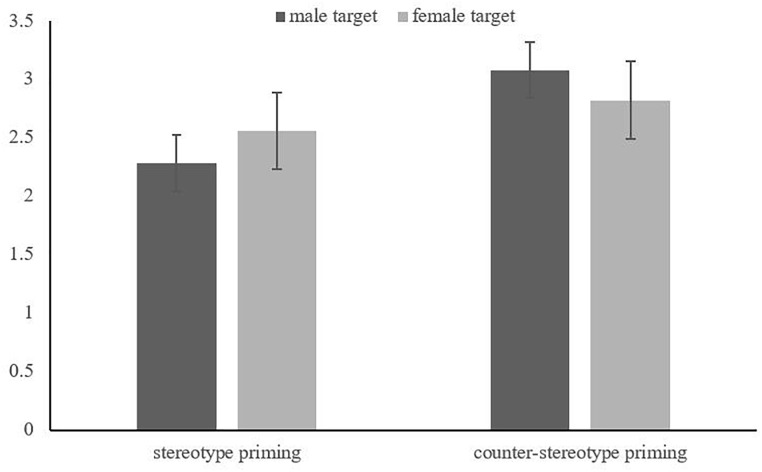
The interaction of target gender and priming type in Experiment 1.

Then, a two-way ANOVA only showed a marginal significant main effect of stereotype priming, *F*(1, 44) = 3.43, *p* = 0.074, η_p_^2^ = 0.071. Specifically, the creativity of stereotype priming conditions (*M* = 2.42, *SD* = 1.06) was lower than that of counter-stereotype priming conditions (*M* = 2.95, *SD* = 0.94). Furthermore, we separated our files by the target’s gender to examine whether different counter-stereotype exemplars have different promotional effects in creativity. Interestingly, the results revealed that counter-stereotype information only promoted creativity when presented a male counter-stereotype exemplar (e.g., male nurse), *F*(1, 22) = 5.36, *p* < 0.05, η_p_^2^ = 0.196, rather than a female counter-stereotype exemplar (e.g., female governor), *F*(1, 22) = 0.42, *p* = 0.316, η_p_^2^ = 0.014. On the other hand, we did not find a significant main effect based on the target’s gender, *F*(1, 44) = 0.00, *p* = 0.981, η_p_^2^ = 0.000, or interaction between independent variables, *F*(1, 44) = 0.88, *p* = 0.359, η_p_^2^ = 0.019 (as shown in Figure 1).

### Discussion

The results of Experiment 1 replicated what previous studies have found (Gocłowska et al., 2013, 2014), in that priming of counter-stereotypes boosted creativity to a certain extent. However, considering differences between the background of Eastern and Western cultures, perhaps the poster design itself was a difficult task for Chinese students since most of them have never previously taken part in a party. Therefore, we decided to transform the measurement of creativity in accordance with our cultural background, which is better reflected in the Chinese Idiom Riddle Test (Zhu et al., 2009). Despite there being insignificant interactions between stereotype priming and the target’s gender, we still found differences in creativity promotion for male and female counter-stereotype exemplars. Counter-stereotypic male exemplars promoted creativity performance more than counter-stereotypic female exemplars. This result is in accordance with a previous finding that people have less tolerance to counter-stereotypic male exemplars (Signorella and Liben, 1984; Hughes and Seta, 2003; Sullivan et al., 2018), implying that this effect may not only generate negative attitudes, but also influence relative cognitive processes.

## Experiment 2: the Mediating Effects of Emotion and Cognitive Flexibility

### Methods

#### Participants

We recruited 104 college students in Wuhan as participants. One of the participant’s information on gender and age was lost; we assigned this individual to the male counter-stereotype priming group. Considering we did not take participant’s gender into account as an independent variable, this participant was still included in our final analysis. Similar to Experiment 1, all the participants were randomly assigned to four experimental conditions and their grouping and age distribution are displayed in Table 2. Participants volunteered to be involved in this study. The informed consent procedure was identical to Experiment 1.

**Table 2:** Participants’ allocation and their age distribution in Experiment 2.

	Stereotype priming group			Counter-stereotype priming group		
Targets’ gender	*N*	*M_age_*	*SD*	*N*	*M_age_*	*SD*
Male	29	19.14	2.15	25	19.16	1.60
Female	23	19.36	2.34	27	18.96	1.61


#### Measurements

For the measurement of creativity, we adopted the Chinese idiom riddle test with a 10-item idiom riddle (Zhu et al., 2009). This test is one of several insight problem-solving tasks developed from traditional Chinese idioms, and its items and options have been examined in previous studies (Zhu et al., 2009; Huang et al., 2013). Each Chinese idiom riddle item was followed by four options: two irrelevant options, one creative option, and one common option. Participants were required to choose a creative answer which they thought would have the same meaning with the riddle item. A participant’s creativity was calculated based on the number of correct selections, with more creative answers indicating a higher level of creativity.

To explore the mediating effect of emotion, especially surprise and delight, participants needed to rate the intensity of their emotions on the 7-point Likert scale (-3 = very unsurprised/un-delighted; 3 = very surprised/delighted). A higher score indicated a more intensive emotion activated by stereotype/counter-stereotype priming.

The measurement of cognitive flexibility was developed from a scale examined by Martin and Rubin (1995). This scale consists of 12 items, including 4 reversed items (2, 3, 5, and 10). Participants were required to rate each item on a 7-point Likert scale (1 = very incongruent and 7 = very congruent). After reversing the scores of these four items, we calculated the mean of all the items as the score of the participant’s cognitive flexibility. In this study, the Cronbach’s α of this scale was 0.83, indicating an accessible reliability of this scale.

#### Procedures

Design of Experiment 2 was similar to Experiment 1, also adopting a 2 (targets’ gender: male vs. female) × 2 (priming type: stereotype vs. counter-stereotype) randomized block design. The dependent variable was the participant’s performance on the Chinese idiom riddle test.

The procedure of Experiment 2 was almost identical with that of Experiment 1, except for creativity and the mediation variables measurements. First, participants were instructed to complete a description task to prime stereotypes/counter-stereotypes. Then they needed to report the typicality of the targets in the description task. A Chinese idiom riddle test followed with a 2-min time restriction. In addition, after measuring the independent and dependent variables, the participants were required to rate their intensity of surprise and delight upon completion of the description task. Finally, participants needed to complete a 12-item cognitive flexibility scale.

### Results

#### The Role of Counter-Stereotypes in Creativity Promotion

We checked the effectiveness of manipulations of priming types. An independent-samples *t*-test showed that the typicality of stereotype priming groups (*M* = 3.29, *SD* = 2.19) was significantly lower than that of counter-stereotype priming groups (*M* = 4.29, *SD* = 1.60), *t*(91.42) = 2.62, *p* < 0.05, *d* = 0.51, *95%CI* = [0.24, 1.75]. Thus, the manipulation of priming type was proven effective.

**FIGURE 2:**
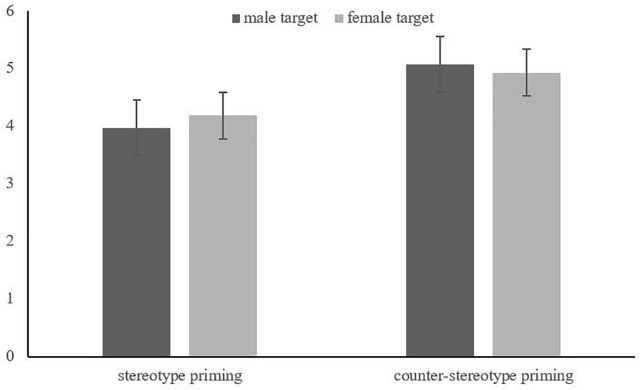
The interaction of targets’ gender and priming type in Experiment 2.

A two-factor ANOVA on participants’ creativity showed a main effect of priming type, *F*(1, 100) = 3.93, *p* = 0.05, η_p_^2^ = 0.04, while there was no main effect of targets’ gender, *F*(1, 100) = 0.01, *p* = 0.95, η_p_^2^ = 0.000; the interaction between priming type and targets’ gender was not significant, *F*(1, 100) = 0.15, *p* = 0.70, η_p_^2^ = 0.002. Compared with stereotype priming (*M* = 4.06, *SD* = 2.59), the counter-stereotype priming (*M* = 5.00, *SD* = 2.10) had better effects on the promotion of participants’ creativity. Further analysis showed that, although both insignificant, male counter-stereotype priming, *F*(1, 53) = 2.68, *p* = 0.11, η_p_^2^ = 0.05, could promote participants’ creativity performance better, than female counter-stereotype priming, *F*(1, 47) = 1.38, *p* = 0.25, η_p_^2^ = 0.03 (as shown in Figure 2).

#### Examination of the Mediating Effects of Emotion and Cognitive Flexibility

We analyzed the mediating effects of emotion (i.e., delight and surprise) and cognitive flexibility using the Process procedure in SPSS (Hayes, 2013). We assumed that all three mediated the influence of priming type on creativity. The descriptive statistics were conducted first, as shown in Table 3.

**Table 3:** Means, *SD*s and correlation of mediate model.

Variables	*M*(*SD*)	Priming type	Surprise	Delight	Cognitive flexibility
Surprise	–0.67 (2.17)	0.42***			
Delight	0.87 (1.85)	–0.03	0.08		
Cognitive flexibility	4.57 (0.80)	0.18	0.09	–0.05	
Creativity	4.51 (2.39)	0.20*	0.09	0.15	0.23^∗^


*Note: ^∗∗∗^*p* < 0.001, ^∗∗^*p* < 0.01, ^∗^*p* < 0.05, ^+^*p* < 0.10.*

As shown in Table 3, there was a significant correlation between priming type and creativity. Hence, we were able to further analyze any mediating effects. The priming types were independent variables, participants’ creativity were dependent variables, while delight, surprise, and cognitive flexibility were mediating variables. The results of the mediating effects are shown in Table 4.

**Table 4:** The mediating effect analysis of emotions and cognitive flexibility.

Predictors	Outcome variables	*β*	*SE*	*t*	*p*
Direct effect					
Priming type	Creativity	0.94	0.46	2.04	0.04
Indirect effect					
Priming type	Cognitive flexibility	0.29	0.16	1.87	0.07
	Surprised	1.796	0.39	4.62	<0.001
	Delighted	–0.083	0.29	–0.28	0.78
Cognitive flexibility	Creativity	0.611	0.29	2.11	0.04
Surprise		–0.017	0.12	–0.15	0.88
Delight		0.269	0.15	1.75	0.08
Priming type		0.819	0.51	1.62	0.11
*R^2^*		0.045			
*F*		4.16^+^			


**FIGURE 3:**
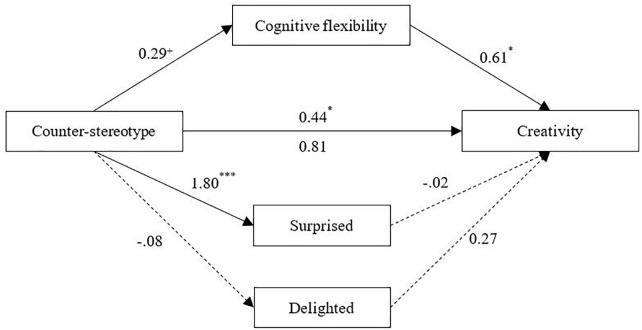
The mediating effect of cognitive flexibility. ^∗∗∗^*p* < 0.001, ^∗∗^*p* < 0.01, ^∗^*p* < 0.05, ^+^*p* < 0.10.

From the results of the mediating effects, we can see a significant direct relationship between predictive variables and explanatory variables, with an accessible overall explanatory power of the model. The priming type could significantly predict participants’ surprise. Meanwhile, its prediction on cognitive flexibility was marginally significant. Furthermore, after including all the mediating variables into our model, we found an attenuated direct relationship between priming type and creativity, which turned out to become insignificant. On the other hand, only participants’ cognitive flexibility could predict their creative performance. Therefore, cognitive flexibility partly mediated the influence of counter-stereotype priming on creativity, as shown in Figure 3.

### Discussion

Experiment 2 replicated the role of counter-stereotypes on creativity promotion. Moreover, it demonstrated a more important pathway of cognition, which played a significant mediating role in this process. Cognitive flexibility partly mediated the relationship between counter-stereotypic priming and creativity, while the mediating effects of emotion (i.e., surprise and delight) were not significant. Compared with stereotype priming, the participants’ cognitive flexibility improved only under counter-stereotype priming conditions, and they performed better on breaking existing mindsets. In addition, the increased cognitive flexibility improved participants’ performances in the following creativity test. This study provides further support for the findings of Gocłowska et al. (2013), confirming their inferences about their results. Researchers have found that people tend to overly rely on their stereotypes and newly-activated knowledge to unconsciously limit their flexible thinking (Ward, 2007). Once provided with the opposite information, their stereotyped or schematized knowledge is no longer effective, and they have to turn from conventional knowledge to newer strategies. In so doing, they construct solutions to problems in a more flexible way, thus demonstrating a higher level of creativity.

## General Discussion

To explore the influence of counter-stereotypes on creativity and its psychological mechanism in a Chinese cultural context, three experiments were designed in this study to verify the relationship between these variables and—for the first time—compared the emotional and cognitive pathways in which counter-stereotypes affect creativity performance. The results demonstrated that in the context of Chinese culture, counter-stereotypes could improve individual creativity while cognitive flexibility played a partial mediating role. The mediating role of surprise and delight was not evident. This study is significant for understanding counter-stereotypes, creativity, and the relationship between the two. First, our replicated results of previous research have demonstrated that the influence of counter-stereotypes on creativity has cross-cultural stability. Second, our study found that creativity performance could be changed through manipulation of counter-stereotypes, which is consistent with previous research and indicates the malleability of creativity (Dumas and Dunbar, 2016). Finally, the confirmation of the mediating role of cognitive flexibility on the relationship between counter-stereotypes and creativity further reinforces and deepens the findings of Gocłowska et al. (2013).

### Cognitive Factors in the Influence of Counter-Stereotypes on Creativity

One of the purposes of this study was to compare the emotional and cognitive pathways through which the counter-stereotype priming affects creativity performance. We found that individual cognitive flexibility can play a partial mediating role in the relationship between counter-stereotypes and creativity. This finding complements previous research on cognitive flexibility (Gocłowska et al., 2014). Although previous research has explored the influence of counter-stereotypes at the cognitive level, most of the research only focused on some concepts in the domain of social cognition (Dasgupta and Greenwald, 2001; Lai et al., 2014; Finnegan et al., 2015). Our present study extends the influence of counter-stereotypic information to a new field, creativity, to which its relevance was previously considered less obvious.

From the perspective of social cognition, we can discover the connection between counter-stereotypic information and creativity. As a simple and quick cognitive schema, stereotypes are key to our social processing. Although this stereotyped way of thinking is fast and effective, it is prone to form an overly-rigid mindset and conflicts with the core process of creativity – the generation of novel connections (Sternberg, 2006; Zhan et al., 2015; Gong et al., 2016). Cognitive-oriented researchers believe that, in our problem-solving process, there are particular scripts which lead to creative thinking (Galinsky et al., 2008). This procedure is also applicable in terms of the influence of counter-stereotypic information. Counter-stereotypic information improves an individual’s cognitive flexibility so that they are no longer limited to existing mindsets, knowledge, and experience. With its influence, people unconsciously pay more attention to novel stimulation, regardless of existing knowledge and mindsets, so that they demonstrate a higher level of creativity.

As a new strategy, the improvement of counter-stereotypy on creativity has practicality. Previous research has suggested that diversified experiences (Godart et al., 2015) or counter-stereotypic information presentation has a positive effect on individuals’ creativity (female engineers; Anderson et al., 2014). This is also implicated in our research. If people can actively and voluntarily enrich their experience or access more counter-stereotypic information, they will improve their creativity even when living in a common environment.

### Emotional Factors in the Influence of Counter-Stereotypes on Creativity

To investigate the mediating effects of surprise and joy, Experiment 2 conducted emotional measurements after priming of stereotype and counter-stereotype information. Even though the results replicated previous studies (Prati et al., 2015), that counter-stereotypes indeed trigger surprise and delight, our results also predicted participants’ creativity. However, the mediating effect of either delight or surprise was not significant, which is probably due to the following reasons.

Firstly, from the perspective of the priming task, existing research has suggested the influence of emotion on creativity through cognitive flexibility and persistence (Nijstad et al., 2010). Thus, in our study, cognitive flexibility relates creativity more directly compared with emotions, because we used the adjective description task for counter-stereotype priming. This task required adequate cognitive processing when participants described the targets. In addition, the Chinese idiom riddle test in this study is a type of insight test, which demonstrates automatic association—through unconsciousness—to creative thinking. This measurement relates more with people’s existing knowledge about traditional Chinese idioms and memory, which is more likely reflecting people’s cognitive abilities. This experiment shows the “matching effect” between counter-stereotype priming tasks and creativity measurements, only connected through cognitive pathways. Therefore, future research could examine whether the promotion of counter-stereotypic information on creativity also acts via emotional pathways, with a more emotional priming approach.

From another perspective, the emotional change due to counter-stereotype priming is momentary. As a result, although the real-time measurement shows that counter-stereotype indeed has triggered the participants’ surprise, this emotional arousal might not be maintained throughout the creativity measurement. Therefore, follow-up studies could try to change the method of stereotype priming and make the priming process produce a more lasting and profound emotional experience.

### The Effect of Counter-Stereotypes on Creativity Differs Across Target’s Gender

Throughout two experiments, we have found a stable difference of creativity promotion between male and female counter-stereotype targets. Male counter-stereotype target improve creativity significantly more than female counter-stereotype target both in poster design and Chinese idiom riddle test.

These results are in accordance with previous findings, which revealed that people have different attitudes toward counter-stereotypic behaviors of male and female (Hughes and Seta, 2003; Signorella and Liben, 1984; Sullivan et al., 2018), i.e., people tend to evaluate negatively to counter-stereotype male rather than counter-stereotype female. This predisposition seems influence subsequent cognitive performance from the view of our study. There are two ways can explain how it works. On the one hand, although the manipulation of stereotype priming was effective in our study, the typicality of counter-stereotype male and female tend to be different. Counter-stereotypic male (i.e., male nurse) was perceived less typical than counter-stereotypic female (i.e., female governor), and it further influence their effect on creativity promotion. On the other hand, because of the negative attitude toward counter-stereotype males, there are less males behaving in counter-stereotypic way in our daily life. Thus, the imagining of a counter-stereotype male (i.e., male nurse) intrigues emotions with higher intensity, as well as a higher level of cognitive flexibility. Thereby improve participants’ creativity.

As mentioned earlier, counter-stereotypes are cognitive views of a social group, which behaviors or traits are contrary to the mindset of its superordinate group (Liu and Zuo, 2006; Leicht et al., 2017). Our findings suggest that the cognitive process of counter-stereotype not only influences the perception and evaluation of a certain group, but also other cognitive functions related, such as cognitive flexibility and creativity. Based on these results, further study can explore whether the typicality of counter-stereotype target mediate the effect of counter-stereotype on creativity, and if counter-stereotype priming has influence on other cognitive functions or processes.

### Research Limitations and Future Directions

Based on previous research, this study demonstrated the correlation between counter-stereotypes and creativity and also examined the mediating role of cognitive flexibility in this process. However, there are still several limitations in our study.

Regarding of the research content, although we only identified the mediating role of cognitive flexibility, there is probably a more complicated underlying psychological mechanism between counter-stereotypes and creativity working through the pathway of cognition and emotion. Future studies can use various paradigms to replicate this effect and explore whether there is a “matching effect” between counter-stereotype priming tasks and creativity measurements. Also, other mediating factors in the influence of counter-stereotype on creativity are needed to be discovered. Furthermore, future research can explore the long-lasting effects of creativity promotion from the perspective of social cognition, which may shed light on developing new ways of creativity training.

## Conclusion

This study adopted different measurements of creativity, both via the poster design and Chinese idiom riddle test. By comparing the participants’ creativity performance in stereotype and counter-stereotype priming conditions, we investigated the internal mechanism of counter-stereotypic information priming on creativity to further explore whether emotions (i.e., delight and surprise) or cognitive flexibility played a mediating role. This study revealed that counter-stereotype priming can significantly improve individual creativity performance, while cognitive flexibility plays only a partial mediating role in this process. Our findings suggest a diversified environment might impact our cognitive process unconsciously, and further beneficial our creative performance.

## Author Contributions

BZ and FW conceived and designed the whole experiments, and wrote the article. MW and YW collected the data.

## Conflict of Interest Statement

The authors declare that the research was conducted in the absence of any commercial or financial relationships that could be construed as a potential conflict of interest.
